# Dual inhibition of EGFR and PI3Kinase signaling in EGFR amplified triple negative breast cancer cells induces apoptosis and reduces tumor growth

**DOI:** 10.1101/2025.06.03.657674

**Published:** 2025-06-07

**Authors:** David J. Wisniewski, Donna Voeller, Yonit A. Addissie, Sachin Kumar Deshmukh, Sharon Wu, Maryam B. Lustberg, Darawalee Wangsa, Danny Wangsa, Kerstin Heselmeyer-Haddad, George W. Sledge, Stanley Lipkowitz

**Affiliations:** 1 Women’s Malignancies Branch, Center for Cancer Research, National Cancer Institute, Bethesda MD; 2 Caris Life Sciences, Phoenix AZ; 3 Yale Cancer Center, New Haven CT; 4 Genetics Branch, Center for Cancer Research, National Cancer Institute, Bethesda MD

**Keywords:** epidermal growth factor receptor amplification, PI3Kinase pathway mutations, triple negative breast cancer

## Abstract

****Background**:**

Epidermal Growth Factor Receptor (EGFR) family signaling is commonly dysregulated in cancer by amplification or activating mutations. We investigated the incidence of EGFR amplification with or without PI3Kinase pathway mutations in breast cancer and whether patients with genetic abnormalities in both pathways can be targeted by dual EGFR/PI3K inhibition.

**Methods:**

EGFR amplification and PI3K pathway mutations were studied through data sets from cBioPortal and Caris. We used the triple negative breast cancer (TNBC) cell lines BT20 (amplified EGFR, PIK3CA activating mutation), MDA-MB-468 (amplified EGFR, PTEN deletion), and MDA-MB-231 (no amplification of EGFR or PI3K pathway mutations). EGFR amplification was determined by immunoblot and fluorescent in situ hybridization, and PI3K mutations by sequencing. Signaling was determined by immunoblot, drug synergy by cell viability, cell death by propidium iodide staining, cell cycle analysis by flow cytometry, and animal studies through xenografts.

**Results:**

EGFR amplification is found in approximately 1–2.5% of breast cancer patients, more frequently in TNBC (2.45–6.7%) and ER-/HER2+ (1.3–6.5%) breast cancers, and in the molecular basal (2.33–8.1%) and HER2 enriched (1.87–5.4%) subtypes. Overall survival is shorter among patients compared to those with unamplified EGFR. Up to 71% of EGFR amplified tumors have activating mutations in the PI3K pathway. Combination of EGFR and PI3K inhibitors more dramatically reduced mTOR and AKT signaling in the BT20 and MDA-MB-468 cells, whereas the inhibition of downstream signaling was less significant in MDA-MB-231 cells. Combination of EGFR and PI3K inhibitors reduced cell viability in these three cell lines, but inhibition was greater, statistically significant, and synergistic in MDA-MB-468 and BT20 compared to MDA-MB-231. Only MDA-MB-468 and BT20 cells had an increased fraction of apoptotic cells. EGFR or PI3K inhibition alone in a BT20 xenograft model reduced tumor volume, however the combination was the only statistically significant reduction in tumor volume when compared to vehicle control.

**Conclusions:**

EGFR/PI3K inhibitor combination causes apoptosis and reduction in tumor growth in cells with EGFR amplification and PI3K alteration. Dual inhibition of EGFR/PI3K presents as a potential targeted therapy in patients with EGFR amplification and aberrant PI3K signaling.

## Background

The ErbB family is comprised of four receptor tyrosine kinases, Epidermal Growth Factor Receptor (EGFR), HER2 (ErbB2), HER3 (ErbB3) and HER4 (ErbB4). Typically, upon ligand binding, the receptors either hetero or homodimerize which allows them to activate downstream signaling. Of note, HER2 does not bind any known ligands but instead is activated by forming hetero or homodimers with other ligand bound EGFR family members (1). EGFR is typically activated upon binding of a ligand such as epidermal growth factor (EGF), resulting in EGFR homodimerization (or heterodimerization with other EGFR receptor tyrosine kinases), and trans-phosphorylation of multiple tyrosine residues on the receptors. Upon activation, EGFR signals through various downstream pathways, including phosphoinositide 3-kinases (PI3Ks) or mitogen-activated protein kinases (MAPKs), resulting in increased proliferation, inhibition of apoptosis, migration, invasion, and other pro-tumor signaling (2–5).

Mutation or overexpression of these family members are common drivers of various cancer types (6). For example, HER2 amplification is a driver in approximately 12–20% of breast cancers, correlates with poor survival, and has been effectively targeted to improve outcomes in patients with HER2 amplified breast cancer (7–10). HER2 activating mutations in the tyrosine kinase domain are found in 2–3% of breast cancers and are likely to behave similarly to HER2 amplification, and HER2 mutants are known to dimerize with EGFR, and induce phosphorylation of EGFR (11).

EGFR can be aberrantly activated through mutations or amplification/overexpression, which drives pro-tumor effects in cancers such as non-small cell lung cancer (NSCLC), pancreatic cancer and head and neck cancer (2, 3, 12–15). Due to the driving effect of EGFR on cancer progression, various therapies have been approved for use in patients with EGFR abnormalities, such as the tyrosine kinase inhibitors (TKI’s) gefitinib, erlotinib, afatinib, dacomitinib, osimertinib, and vandetanib, and the monoclonal antibodies cetuximab, panitumumab and necitumumab (2). EGFR has been shown to be expressed in basal triple negative breast cancer (TNBC), however there are various reports which define the degree of EGFR overexpression differently with varying methods (16). In general, EGFR has some degree of elevated expression by immunohistochemistry in breast cancer samples (17–23). Further, EGFR expression was associated with tumor size, poor differentiation, worse relapse-free and overall survival, and shorter metastasis free survival (24, 25). Depending on the study, EGFR gene amplification has been observed in 0.8–14% of breast cancer patients and is associated with high grade but has not always been associated with survival (20, 26–28). Lastly, mutation of EGFR in breast cancer can range from approximately 2–11% (22). Relatively small studies looking specifically at metaplastic breast carcinomas have observed amplification of EGFR in 33–37% of patients (29, 30). Despite these data, clinical trials investigating EGFR inhibitors have not shown great promise in breast cancer, with studies finding partial response rates of 0–6% (18, 20, 22, 31, 32). It is important to note that these studies were typically in heavily pretreated patients, and patients were not stratified by EGFR amplification status. Therefore, investigation of combination therapy targeting EGFR and other pathways may elucidate the functional benefits of targeting a subset of breast cancer patients with EGFR amplification and provide insight into optimal therapy.

In this study we evaluated the frequency and subtype distribution of EGFR amplification and found that EGFR is amplified in approximately 1–2.5% of breast cancer cases, with the most frequent amplification in TNBC, ER-, and ER-/HER2+ tumors, HER2 enriched, basal, and normal subtypes, depending on the dataset studied. Further, we found that up to 71% of EGFR amplified breast cancers had an activating alteration of the PI3K pathway. Using TNBC models of EGFR amplification and PI3K alteration, we demonstrate that dual inhibition of EGFR and PI3K significantly reduced P70S6K, AKT and S6 signaling and cell viability. Further, dual inhibition caused G1 accumulation, and reduction in S phase and G2/M phase, with a caspase-dependent increase in cancer cell death. Lastly, dual inhibition statistically significantly reduced tumor volume *in vivo* as compared to control.

## Methods

### Materials

Z-VAD-FMK (S7023), Erlotinib (S7786), BKM120 (S2247), Afatinib (S1011), Alpelisib (S2814) and sodium carboxymethyl cellulose (S6703) were purchased from Selleck Chemicals. Fetal bovine serum (FBS) (631106) was obtained from Takara Bio. 4–20% precast polyacrylamide gels (catalog #5671094) were purchased from Bio-Rad. Immobilon PVDF transfer membrane (IPVH00010), mini protease inhibitor cocktail (11836153001) and propidium iodide (P4864) was purchased from Millipore-Sigma. PBS (21–031-CV), EGF (354042) and Phenol Red-Free LDEV-Free Matrigel (356237) was purchased from Corning. Sodium orthovanadate (P0758S) was from Fisher Chemicals. NP40 Cell Lysis Buffer (FNN0021), Penicillin-Streptomycin (15140–122), tissue extraction reagent (FNN0071) and RPMI 1640 medium (11875–093) was obtained from ThermoFisher Scientific. were purchased from Selleck Chemicals (Houston, TX).

### Antibodies

Anti-phospho-HER3 Y1289 (4791S), anti-HER3 (12708S), anti-EGFR (2232), anti-PTEN (9559S), anti-phospho-EGFR Y1068 (2234), anti-phospho-AKT S473 (9271S), anti-AKT (9272S), anti-phospho-MAPK T202/Y204 (9101), anti-phospho-p70S6K T389 (9234S), anti-p70S6K (9202S), anti-phospho-S6 S235/236 (4856S), anti-S6 (2217S) and anti-PI3K (4249S) were purchased from Cell Signaling Technology. anti-HSC70 (sc-7298) and anti-ERK2 (D-2, sc-1647) antibodies were purchased from Santa Cruz Biotechnology. Goat Anti-Mouse IgG HRP conjugate (172–1011) and Goat Anti-Rabbit IgG HRP conjugate (1721019) were purchased from BioRad.

### Cell Lines

MDA-MB-468, BT20, MDA-MB-231 were obtained from ATCC, were maintained in RPMI 1640 medium with 10% FBS and 1% penicillin/streptomycin, and grown at 37°C, 5% CO_2_.

### Caris dataset analysis

#### Next-generation sequencing methods (WES, Hybrid)

Because this study utilized samples spanning several years, the methods (including materials and software) used for DNA and RNA profiling evolved over time. Initially, DNA was profiled using whole exome sequencing (WES) with targeted enrichment of 720 clinically relevant genes in 2020; whole transcriptome sequencing (WTS) was performed separately. In 2023, this approach was replaced by MI Tumor Seek Hybrid, which analyzes RNA and DNA from the same total nucleic acid extraction. Nucleic acid was extracted using appropriate FFPE kits for RNA, DNA, or total nucleic acid. DNA sequencing was performed using by whole exome sequencing with enrichment of 720 clinically relevant genes on the NovaSeq 6000 platform (RRID:SCR_016387) (Illumina, Inc., San Diego, CA). WTS was performed using the Illumina Novaseq 6000 platform to an average of 60M reads. Raw WTS data was demultiplexed by Illumina Dragen BioIT accelerator, trimmed, counted, PCR-duplicates removed, and aligned to human reference genome (hg19/hg38) by STAR aligner (RRID:SCR_004463) (33). For transcription counting, transcripts per million (TPM) molecules were generated using the Salmon expression pipeline (RRID:SCR_017036) (34). For MI Tumor Seek Hybrid, RNA was labeled during first strand cDNA synthesis by adapter sequences on the 5’ end of the cDNA primers and next-generation sequencing was performed (720 clinically relevant genes were sequenced at high-depth). Sequencing data was extracted into split FASTQ files (RNA and DNA) for processing. DNA variants detected were mapped to reference genome hg38, and well-established bioinformatics tools such as Burrows-Wheel Aligner (BWA 0.7.17) (RRID:SCR_010910), SamTools (RRID:SCR_002105), Pindel (RRID:SCR_000560), and snpFF (RRID:SCR_005191) were incorporated to perform variant calling functions; germline variants were filtered with various germline databases, including dbSNP (RRID:SCR_002338). Genetic variants identified were interpreted by board-certified molecular geneticists and categorized as ‘pathogenic,’ ‘likely pathogenic,’ ‘variant of unknown significance,’ ‘likely benign,’ or ‘benign,’ according to the American College of Medical Genetics and Genomics (ACMG) standards. Pathogenic and likely-pathogenic variants are counted as “reportable”. A copy number (CN) cutoff of CN ≥ 6 was used to define gene amplification. The CN cutoff of 6 for amplification was determined internally at Caris as a standard (based on MYC/ERBB2 and validated with IHC).

#### Real-world overall survival analysis

Real-world overall survival (OS) information was obtained from insurance claims data. OS was calculated from time of tissue collection as a surrogate for diagnosis until last contact. Patients without contact/claims data for a period of at least 100 days were presumed deceased. Conversely, patients with a documented clinical activity within 100 days prior to the latest data update were censored in the analysis. Kaplan-Meier estimates were calculated for molecularly defined patient cohorts. Hazard ratios (HR) were determined by Cox Proportional Hazards model and *p*-values by log-rank test. Significance was determined as *p* < 0.05.

### cBioPortal dataset analysis

Datasets from TCGA (Cell 2015), Metabric (Nature 2012 and Nature Communications 2016) and MSKCC (Cell 2018) were searched for EGFR amplification, as well as mutations in PIK3CA, PIK3R1, PIK3R2, PIK3R3, PTEN, AKT1, AKT2, AKT3, RPS6KB1, RPS6KB2 and RPS6 in cBioPortal (cbioportal.org) (35–37). Amplification was defined by GISTIC2.0 score = 2 which corresponds to high levels of amplification (copy number > 5) (38).

### Western Blot

Cell lysates were collected, prepared and immunoblotted as previously described (39). Every immunoblot was repeated at least 3 times, with densitometric analysis of band intensities being calculated using ImageJ. Data were presented as an average ± SEM.

### Fluorescence *in situ* Hybridization (FISH)

To prepare metaphase chromosomes, cells were treated with 0.02 mg/ml Colcemid (Invitrogen, Grand Island, NY) for one hour, suspended in a hypotonic solution for 20 minutes, and then fixed using a methanol/acetic acid mixture (1:3 ratio). The cell suspension was dropped onto slides in a cytogenetic drying system (Thermotron, Holland, MI).

Dual color FISH was conducted using a contig of three overlapping bacterial artificial chromosome (BAC) clones containing the EGFR gene on chromosome band 7p11.2, labeled in red, and a centromere probe for chromosome 7 (CEP7) labeled in aqua. The EGFR and CEP7 probes were custom manufactured to our specifications by Cytotest (Rockville, MD), with dyes sourced from Dyomics (Jena, Germany).

In brief, the FISH hybridization protocol included incubating the slides in 1× PBS for 10 minutes, followed by treatment in a pre-warmed pepsin solution (final concentration: 0.02 μg/μl pepsin and 0.01N HCl in dH_2_O) at 37°C for 2 to 5 minutes. The slides were then washed in 1x PBS, dehydrated in an ethanol series, and left to air-dry. Co-denaturation was performed at 72°C for 2 minutes, followed by overnight hybridization at 37°C on a ThermoBrite (Abbott Molecular, Des Plaines, IL). The detection protocol involved washing the slides in 2× SSC/0.3% Nonidet P-40 for 2 minutes at 48°C, followed by a wash in 2× SSC/0.1% Nonidet P-40 for 1 minute at room temperature. Finally, an antifade solution (Vector Laboratories, Burlingame, CA) containing the nuclear counterstain 4,6-diamidino-2-phenylindole was applied, and coverslips were added. Images were captured using a Leica DM-RXA fluorescence microscope (Leica, Wetzlar, Germany) equipped with a 40 X objective and custom optical filters (Chroma, Bellows Falls, VT).

### Sanger Sequencing

Sanger DNA sequencing of genomic DNA from the MDA-MB-231, MDA-MB-468 and BT20 cell lines for PIK3CA mutations was performed using the Big Dye Terminator v.1.1 Cycle Sequencing Kit (Applied Biosystems, CA) according to the manufacturer’s specifications and run on an ABI 3130xl or 3730 Genetic Analyzer (Applied Biosystems, CA). Sequencing was conducted at the CCR Genomics Core at the National Cancer Institute, NIH, Bethesda, MD. Primers were synthesized by (Eurofins Genomics) as follows:

exon 9 sequencing primer (AAGATTTGCTGAACCCTATTG);

exon 20 sequencing primer (TCGTCACAATAGTAACATCATGG).

### Cell Death

CytoTox-Glo (G9291) was performed as directed by Promega. Briefly, cells were pretreated with 20 μM ZVAD for 1 hour, followed by co-treatment with DMSO, 10 μM erlotinib, 1 μM BKM120 or the combination for 48 hours. Dead cell percentage was then calculated according to the manufacturer instructions.

### Cell Viability

Viability was determined by CellTiter-Glo 2.0 (Catalog #G9242) as directed by Promega. Briefly, 10,000 cells were plated in a 96-well white walled plate (Corning #3610) and treated the following day with experimental conditions for 48 hours in 1% FBS RPMI. Reagent was added to each well, the plate was shaken for two minutes and then incubated at room temperature for 10 minutes. Luminescence was then recorded using a plate reader.

### Synergy Scores

Calculated using SynergyFinder.org, using a matrix format with data calculated as percent viability. Data was interpreted as previously described (40).

### Cell Death

Cell death was determined by using propidium iodide (PI) staining, imaging and quantification using the BioTek Cytation 1 Imaging Reader from Agilent (Santa Clara, CA). Briefly, 5,000 cells were plated in a 96-well plate, and treated the next day with experimental conditions in 1% FBS RPMI, along with PI (1:3000 dilution). Time 0 images were acquired, and then cells were incubated at normal cell culture conditions for 72 hours, followed by a second image acquisition. Dead cell percentage was calculated by dividing dead cell number (PI stained) by brightfield cell count number and multiplying by 100. Dead cell percentage was normalized relative to the Day 0 dead cell percentage to account for variation among wells. PI staining positive and brightfield cell quantification was optimized and performed in a cell-line specific manner, using Gen5 Image Prime 3.14 software (Agilent). In experiments using ZVAD-FMK, cells were pretreated +/− 20 μM ZVAD-FMK for 1 hour, followed by co-treatment with experimental conditions.

### Cell cycle

Cells were plated in 6-well plates (3×10^5^ cells/well) and treated with EGFR and/or PI3K inhibitors. After 48 hours, cells were harvested, washed with PBS and fixed in 70% ethanol for at least 1 hour at 4°C. Cells were then washed twice with cold PBS after which 50 ul of 100 ug/ml RNAse A and 200 ul of 50 μg/ml propidium iodide were added. After incubation for 1 hour at room temperature, cell cycle data was acquired using a BD LSRFortessa^™^ (BD Biosciences) flow cytometer and analyzed using FlowJo^®^ software (FlowJo LLC).

### Animal Studies

Sixty female athymic nude mice between 5–6 weeks in age were obtained (Charles River Laboratories) and were housed and observed according to approved NCI-ACUC guidelines. 5 million BT20 cells suspended in matrigel (1:1 with PBS) were injected subcutaneously into the mammary fat pad with a 25G needle (Terumo Medical Care Solutions; SS-01T2516). Once the tumors grew to 100 mm^3^ the mice were randomized into four groups of 15. 0.5% sodium carboxymethyl cellulose (CMC) in water (w/v) was used as control, and afatinib or alpelisib were suspended in 0.5% CMC. Control or 20 mg/kg alpelisib or afatinib, or the combination of both was given by oral gavage (1 inch, 22G needle) once a day for five days each week for the duration of the study. After the first four days of treatment, 5 mice per group were sacrificed, tumors were excised and proteins were harvested in Tissue Extraction Reagent I (Invitrogen-FNN0071) according to the manufacturers protocol. Tumor caliper measurements and body weight were measured twice a week for the duration of the study, as required by humane endpoints for all mice. Tumor volumes were calculated according to the formula V = ½ (length × width^2^). All aspects of this study were approved by the NCI Animal Care and Use Committee (IACUC number WMB-004) and performed in accordance with the approved protocol. Maximum tumor size for each animal was 20 mm in any direction, and this maximum tumor size was not exceeded. As soon as tumors reached maximum size, the mice were humanely euthanized.

### Statistical Analysis

Student’s *t*-test with 2 tailed comparisons assuming equal variance, one-way or two-way ANOVA were performed as indicated. P-values of ≤0.05 were considered significant.

## Results

### Incidence of EGFR amplification in breast cancer

**Figure 1.**
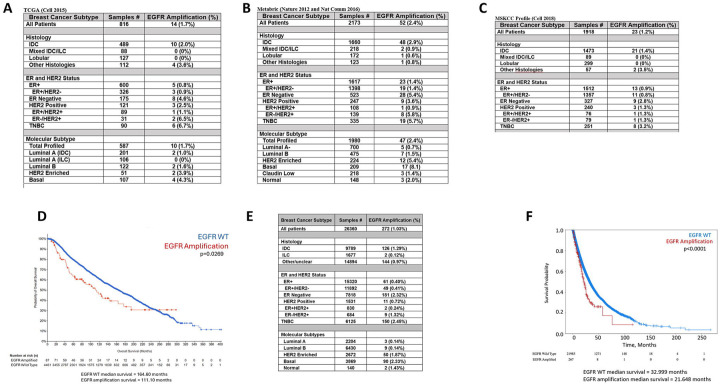
Incidence of EGFR amplification in breast cancer. A) Breast cancer subtype analysis of patients in TCGA dataset with EGFR amplification. B) Similar analysis of METABRIC dataset, and C) MSKCC dataset. D) Combination of A-C datasets to analyze overall survival (OS) of patients comparing EGFR amplification (red line) to patients without EGFR amplification (blue line). E) Breast cancer subtype analysis of patients in Caris dataset with EGFR amplification. F) OS of patients with EGFR amplification (red line) compared to patients without EGFR amplification (blue line).

The frequency of EGFR amplification in breast cancer was evaluated in the TCGA, METABRIC and MSKCC databases in cBioPortal (35–37) . EGFR amplification was observed in 1.2–2.4% of cases, depending on the dataset (Figure 1A–C). The TCGA and METABRIC databases contain primarily early-stage primary breast cancers while the MSKCC dataset is approximately 50% metastatic/recurrent breast cancers. We analyzed EGFR amplification by subtype and found that amplification is more common in invasive ductal carcinomas (IDC) (1.4–2.9%) compared to invasive lobular carcinomas (ILC) (0–0.6%). Further, the highest rate of amplification was in TNBC (3.2–6.7%), ER− (2.8–5.4%) and ER-/HER2+ tumors (1.3–6.5%), whereas EGFR amplification was relatively less common in ER+ breast cancers (0.8–1.4%). Using PAM50 classification, EGFR amplification is the highest in basal (4.3–8.1%) and HER2 enriched (3.9–5.4%) molecular subtypes, compared to luminal A or luminal B subtypes (0.7–1% and 1.5–1.6%, respectively) (41). To determine the prognosis of patients with EGFR amplification, we combined the TCGA, METABRIC and MSKCC datasets due the small number of cases in each and evaluated overall survival (OS) (Figure 1D). There is a statistically significant lower OS in patients with amplified EGFR, with a difference of 53.5 months (111.4 vs 164.6 months) (Figure 1D).

To confirm these data in a larger dataset with clinical annotation, we evaluated the incidence of EGFR amplification in patients with breast cancer profiled by Caris Life Sciences (hereafter referred to as Caris), where the majority of patients profiled had metastatic breast cancer (Figure 1E). EGFR amplification was found in 1.03% of the breast cancers profiled. The lower frequency of EGFR amplification seen in the Caris data set may be due to higher cutoff used for calling amplification, since cBioportal uses a GISTIC2.0 score of 2 to indicate amplification which corresponds to CN ≥4–5 while Caris used a CN ≥ 6 as the cutoff for amplification. As in the other datasets, EGFR amplification was more prevalent in IDC (1.29%) compared to ILC (0.12%). EGFR amplification was more prevalent in TNBC (2.45%), and in the molecular HER2 enriched (1.87%), basal (2.33%) and normal (1.43%) subtypes. In the entire cohort of patients, OS was statistically significantly shorter in patients with EGFR amplification compared to those with wild type EGFR (21.6 vs 33 months; Figure 1F). Overall, this data shows that there is a subset of breast cancer patients with EGFR amplification that is most frequent in TNBC and ER-/HER2 amplified tumors and is associated with a poor prognosis.

Since EGFR amplification is enriched in patients with TNBC or Basal cancers, we investigated the differences in overall survival in patients with EGFR amplification depending on subtype using the Caris dataset (Additional File 1). There was no OS difference in TNBC or Basal cancers with EGFR amplification (Additional File 1 A and B). HER2 enriched patients with EGFR amplification had a trend for a slightly worse overall survival (difference of 6.7 months), however this was not statistically significant (p=0.17) (Additional File 1 C). In contrast, patients with ER+/HER2− tumors had a statistically significant worse OS in EGFR amplified patients (difference 22.9 months; p<0.00001) (Additional File 1 D).

### *In vitro* modeling of EGFR amplification and PI3K alterations

**Figure 2.**
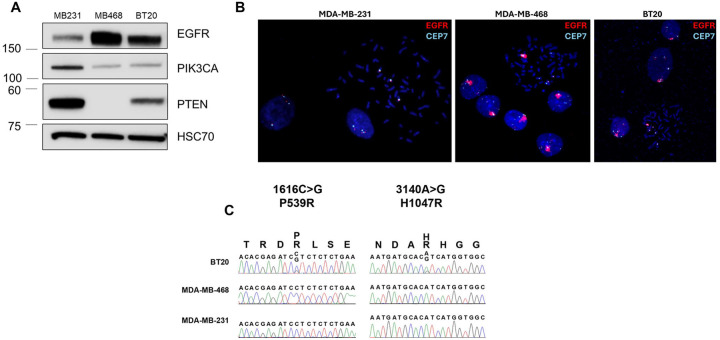
EGFR protein expression and gene amplification, and PI3K mutations in MDA-MB-231, MDA-MB-468 and BT20 cells. A) Western blot showing protein expression of EGFR, PIK3CA, pTEN and HSC70 (loading control) in MDA-MB-231, MDA-MB-468 and BT20 cell lines. B) FISH analysis showing EGFR (red) and CEP7 (aqua, control) in MDA-MB-231, MDA-MB-468 and BT20 cell lines. C) DNA sequencing for PIK3CA mutation status in BT20, MDA-MB-468 and MDA-MB-231 cell lines.

To evaluate the role of EGFR amplification in cell models, we used two cell lines with amplified EGFR (MDA-MB-468 and BT20) and one cell line without amplified EGFR (MDA-MB-231). As evidenced in Figure 2A, MDA-MB-468 and BT20 both have elevated EGFR protein levels as compared to MDA-MB-231. Fluorescence *in situ* hybridization (FISH) analysis confirmed the amplification of EGFR in both MDA-MB-468 and BT20, and the lack of EGFR amplification in MDA-MB-231 (Figure 2B). MDA-MB-468 has loss of PTEN protein, which supports previously published studies showing loss of PTEN expression in these cells (Figure 2A) (42, 43). As previously reported, the BT20 TNBC cell line has two activating mutations in PIKCA (P539R and H1047R), which previously have been shown to be in cis (43–45) (Figure 2C). MDA-MB-468 and MDA-MB-231 have wildtype PIK3CA. Therefore, MDA-MB-468 and BT20 were the two cell lines with EGFR amplification and PI3Kinase pathway activating mutations, and MDA-MB-231 was the control cell line without amplified EGFR or PI3Kinase pathway mutations.

### PI3K pathway mutations in EGFR amplified breast cancer

**Figure 3.**
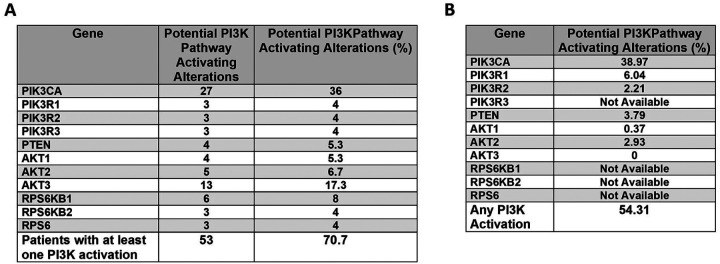
PI3K pathway mutations in EGFR amplified breast cancer. A) Datasets from TCGA, METABRIC and MSKCC showing alterations in PI3K pathway genes in 75 patients with EGFR amplification. B) Dataset from Caris showing alterations in PI3K pathway genes in 272 breast cancers with EGFR amplification.

Considering the co-incidence of mutations or alterations in PI3K signaling in these EGFR amplified cell lines, we investigated how often this occurs in patients. Once again using cBioPortal, in a total of 75 patients with EGFR amplification from the TCGA, METABRIC and MSKCC cohorts, we discovered that approximately 53 patients (71%) with EGFR amplified tumors have at least one genetic alteration which can potentially activate PI3K signaling (Figure 3A and Additional File 2). 36% have an activating mutation in PIK3CA, 5.3% have a loss of PTEN, 12% have mutations in PIK3R1, R2 or R3, and 29.3% have amplification of AKT1, 2, or 3. Also, there are 16% of patients who have amplifications downstream of mTOR (either in ribosomal protein S6 kinase B1 or B2, or ribosomal protein S6). Of note, multiple tumors had two or more alterations in this pathway, which can be observed in the oncoplot of the EGFR amplified tumors (Additional File 2).

Similarly in the Caris dataset, 54.3% of EGFR amplified tumors have mutation or amplification in the PI3K pathway (including PIK3CA, pTEN, PIK3R1, PIK3R2, or AKT 1, 2 or 3) (Figure 3B). The largest percentage of alterations are in PIK3CA (39%). Of note, the Caris dataset does not include alterations in the genes downstream of mTOR, which contributes to the difference comparing the sums of Caris and the cBioPortal data. This data confirms that the majority of tumors with EGFR amplification are likely to have co-incident aberrant PI3K signaling.

### EGFR and PI3K dual inhibition significantly reduce downstream signaling in EGFR amplified and PI3K altered breast cancer

**Figure 4.**
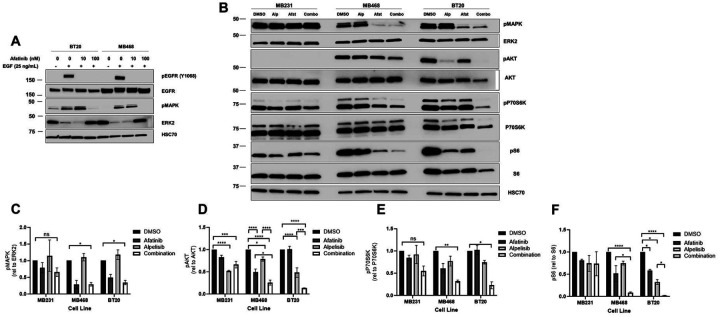
EGFR/PI3K dual inhibition significantly reduces downstream signaling in EGFR amplified and PI3K altered breast cancer. A) BT-20 and MDA-MB-468 cells were pretreated for 24 hours with 0, 10 or 100 nM afatinib, and then starved in serum-free RPM1 media for 3 hours prior to stimulation with or without 25 ng/mL EGF for fifteen minutes. Cells were lysed and immunoblotted for pEGFR (Y1068), EGFR, pMAPK (T202/Y204), ERK2 and HSC70 (loading control). B) MDA-MB-231, MDA-MB-468 and BT20 cells were treated with 100 nM alpelisib (Alp), 1 μM afatinib (Afat) or a combination of both for 24 hours, and were then lysed and immunoblotted for pAKT (S473), AKT, pMAPK (T202/Y204), ERK2, p-p70S6K (T389), p70S6K, pS6 (S235/236), S6 and HSC70 (loading control). In at least three independent experiments, the band density of the phosphorylated protein relative to total protein was averaged ±SEM for C) pMAPK/ERK2, D) pAKT/AKT, E) p-p70S6K/p70S6K and F) pS6/S6. Two-way ANOVA was performed, where ns indicates not statistically significant, * indicates p<0.05, ** indicates p<0.01, *** indicates p<0.001 and **** indicates p<0.0001.

To elucidate the effect of EGFR signaling in these two EGFR amplified cell lines, we treated the cells with epidermal growth factor (EGF) and the tyrosine kinase inhibitor (TKI) afatinib and observed that EGFR phosphorylation was decreased upon afatinib treatment in both MDA-MB-468 and BT20 cell lines, indicating that the drug inhibited EGFR signaling upon EGF stimulation (Figure 4A). We confirmed this observation by using another TKI, erlotinib (Additional File 3 A). To determine the effects of PI3K and EGFR dual inhibition on basal signaling mechanisms, we looked at pathways downstream of EGFR signaling, specifically focusing on MAPK, AKT, p70S6K and S6K signaling. In the EGFR amplified cell lines MDA-MB-468 and BT20, afatinib significantly inhibited basal MAPK signaling, but had no significant effect on MAPK signaling in MDA-MB-231 cells (Figure 4B and C). Therefore, EGFR amplified cells have basally activated MAPK signaling downstream of EGFR. Afatanib alone also down regulated AKT signaling in the MDA-MB-468 cells but not in the BT20 or the MDA-MB-231 cell lines (Figure 4B and D). The PIK3CA inhibitor alpelisib significantly down regulated AKT activation in all three cell lines but did not affect pMAPK (Figure 4B and D, Additional File 3 B). The combination of afatanib and alpelisib led to a greater reduction in in AKT phosphorylation in the EGFR amplified cell lines (MDA-MB-468 and BT20) than either drug alone (Figure 4B and D). MDA-MB-231 cells, which do not have EGFR amplification or PI3K alterations, did not have a significant reduction in AKT signaling when comparing the drug combination to either drug alone, which can be seen with a darker exposure western blot (Figure 4D and Additional File 3 B). All three cell lines had measurable p-p70S6K and an pS6, however the drug combination only elicited a significant difference compared to DMSO control in BT20 and MDA-MB-468 (Figure 4B, E and F). It is noteworthy that afatinib or alpelisib alone in BT20 cells reduced pS6 in a statistically significant manner, however the combination of the two drugs reduced pS6 to near undetectable levels, providing evidence that dual inhibition is the most effective treatment at reducing S6 signaling (Figure 4B and 4F). No significant effects on p-p70S6K or pS6 were seen in MDA MB-231 treated with either drug alone or the combination.

To confirm that this downregulation of MAPK, AKT, p70S6K and S6K signaling was due to a class effect and not these specific inhibitors, we also used EGFR TKI erlotinib and PI3K inhibitor BKM120 and observed similar results (Additional File 3 C-F). Overall, this shows that the inhibition of pMAPK and a synergistic downregulation of pAKT, p-p70S6K and pS6K signaling occurs in response to dual EGFR and PI3K inhibition in cells with EGFR amplification and dysregulated PI3K in response to the dual inhibition of EGFR and PI3K. The greatest effect was observed in BT20 cells which have EGFR amplification and PIK3CA activating mutations.

It has been previously described that EGFR inhibition by cetuximab for 24 hours can increase expression of HER3, leading to heterodimerization of EGFR and HER3, which in turn overrides the EGFR inhibition by cetuximab (46). In our work we did not see changes in HER3 or phospho-HER3 levels after 24 hours of exposure to the TKI erlotinib. Further, the phosphorylation of HER3 was inhibited by erlotinib in both of the EGFR amplified cell lines (Additional File 4). HER3 levels were very low, and phosphorylation was not detected in the MDA-MB-231 cells. It is likely that increased expression of HER3 cannot overcome inhibition by the TKI as it does for cetuximab since the kinase activity of EGFR is necessary for signaling of the EGFR/HER3 heterodimer (47).

### Dual EGFR and PI3K inhibition synergistically reduce cell viability in breast cancer cells with EGFR amplification and PI3K alteration

**Figure 5.**
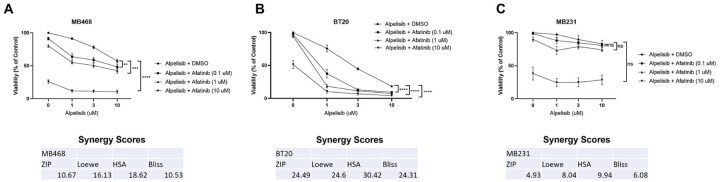
Dual EGFR/PI3K inhibition synergistically reduce cell viability in TNBC with EGFR amplification and PI3K alteration. A) MDA-MB-468, B) BT20 and C) MDA-MB-231 cells were treated with the indicated doses for 48 hours, and then viability was determined by Promega CellTiter-Glo 2.0. Data represents the average ±SEM of at least three independent experiments, and statistical analysis was performed as Two-Way ANOVA, and the p value indicates interaction, where ns indicates not statistically significant, ** indicates p<0.01, *** indicates p<0.001 and **** indicates p<0.0001. The averages were also subjected to four different synergy score analyses through synergyfinder.org, where a score of >+10 indicates synergy, −10 to +10 indicates additive effects, and <−10 indicates antagonism.

Next, we tested the effects of afatinib and alpelisib in combination on cell viability, to determine whether this disruption in signaling had a functional effect. In MDA-MB-468 cells, which have EGFR amplification and PTEN deletion, each drug alone significantly inhibited viability but the combination showed statistical significance when comparing the combination of drugs to either drug alone, and exhibited synergy using all four calculations as determined by SynergyFinder, with values over ten (Figure 5A) (40). In BT20 cells, which have EGFR amplification and PI3K mutations, the individual drugs again significantly inhibited viability and the combination demonstrated the most significant effect of synergy with the drug combinations completely inhibiting cell viability, as exemplified by the highest synergy score values (Figure 5B). In MDA-MB-231 cells, which do not have EGFR amplification or PI3K alterations, there was not significant viability inhibition by alpelisib alone, and there was only an inhibition of viability to a high dose of afatinib (Figure 5C). The combination of the two drugs did not significantly affect viability, and synergy scores calculated resulted in values indicating an additive effect of the two drugs together (score between −10 and 10) (Figure 5C). Overall, EGFR and PI3K inhibitors in combination exhibit a synergistic effect on cell viability in cell lines with EGFR amplification and PI3K alterations, and only an additive effect in a cell line without EGFR amplification or PI3K alterations.

We confirmed these effects to be a class effect and not a drug specific effect by testing the combinations of erlotinib and BKM-120 (Additional File 5 A-C). Erlotinib reduced the viability of all three cell lines, however there is a much more significant decrease in cell viability in the two EGFR amplified cells. When combined with BKM120, there is a further reduction of cell viability in the EGFR amplified cells. Specifically, in BT20 cells, a combination of erlotinib and BKM120 resulted in 90% reduction in cell viability. Overall, this data confirms that EGFR amplified and aberrant PI3K cell lines MDA-MB-468 and BT20 have an increased reliance on EGFR and PI3K signaling for cell viability regardless of EGFR TKI or PI3K inhibitor.

### Dual EGFR and PI3K inhibition induce cell cycle arrest and apoptosis in EGFR amplified and PI3K altered breast cancer cells

**Figure 6.**
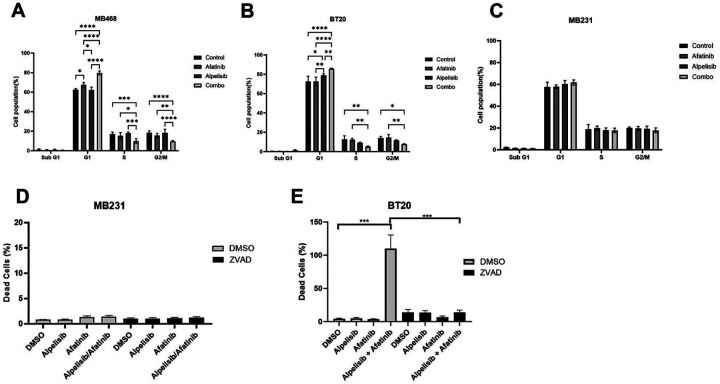
EGFR/PI3K inhibition induce cell cycle arrest and apoptosis in EGFR amplified and PI3K altered TNBC. A) MDA-MB-468 were treated with DMSO (control), 1 μM afatinib, 10 μM alpelisib, or the combination of both B) BT20 were treated with DMSO (control), 100 nM afatinib, 1 μM alpelisib, or the combination of both and C) MDA-MB-231 were treated with DMSO (control), 1 μM afatinib, 10 μM alpelisib, or the combination of both for 48 hours, and then were harvested, washed, fixed and stained with propidium iodide before being analyzed by flow cytometry and FlowJo for cell cycle changes. Data represents the average ±SEM of at least three independent experiments, and Two-Way ANOVA statistical analysis was performed where * indicates p<0.05, ** indicates p<0.01, *** indicates p<0.001 and **** indicates p<0.0001. D) MDA-MB-231 and E) BT20 cells were pretreated with 20 μM Z-VAD-FMK (ZVAD) for one hour, and then were treated with doses DMSO, 1 μM alpelisib, 1 μM afatinib, or the combination of alpelisib and afatinib with continued ZVAD treatment in 1% FBS RPM1 media containing propidium iodide for 72 hours. Imaging of the cells was performed by BioTek Cytation 1, and dead cell percentage was calculated by dividing PI positive cells by the total brightfield cell count and multiplying by 100. Data represents the average ±SEM of at least three independent experiments. Statistical analysis was performed by Student’s t-test, with *** indicating p<0.001.

To evaluate the reason for the reduction in cell viability, we performed cell cycle analysis and observed that there is an increase in cells in the G1 phase of the cell cycle when EGFR and PI3K are inhibited by the combination of alpelisib and afatinib in both MB468 and BT20 cells (Figure 6A and B). In addition to the G1 accumulation, the drug combination also exhibited statistically significant reductions in both the S phase and G2/M cell populations. Further, cell cycle was not significantly altered in MB231 cells which do not have EGFR amplification or PI3K alteration (Figure 6C). EGFR and PI3K inhibition with erlotinib and BKM120 combination had similar effects on cell cycle, however there was also a statistically significant increase in sub-G1 population with the drug combination in both MDA-MB-468 and BT20, however there was no effect in control MDA-MB-231 (Additional File 5 D-F).

To determine whether EGFR and PI3K inhibitor combinations induced cell death and apoptosis, we quantified dead cell percentages using propidium iodide (PI) staining of cells treated with afatanib, alpelisib, and the combination in the presence or absence of Z-VAD-FMK (ZVAD), which is a pan-caspase inhibitor. In control MDA-MB-231 cells, drugs alone or in combination did not show an increase in dead cell percentage, consistent with our viability data showing a lack of synergy for inducing cell death with these drugs in cells without EGFR amplification and PI3K mutation (Figure 6D). In contrast, in the BT20 cells with EGFR amplification and PI3K mutation, the combination of afatanib and alpelisib resulted in a dramatic, statistically significant increase in dead cells while each drug alone did not induce cell death (Figure 6E). Pre-treatment of the cells with ZVAD blocked this death, showing that the cell death observed is caspase dependent. We similarly confirmed that induction of cell death was caspase-dependent in BT20 and MDA-MB-468 with erlotinib and BKM-120 being rescued by ZVAD treatment (Additional File 6). It should be noted that erlotinib and BKM-120 alone did increase caspase-dependent cell death, however the greatest and most significant increases in cell death were when the drugs were combined. The MDA-MB-231 cells only showed a slight increase in cell death with each erlotinib alone and no further increase in the effect of the combination when compared to erlotinib alone (Additional File 6).

### Dual EGFR and PI3K inhibition is most effective at reducing tumor volume *in vivo*

**Figure 7.**
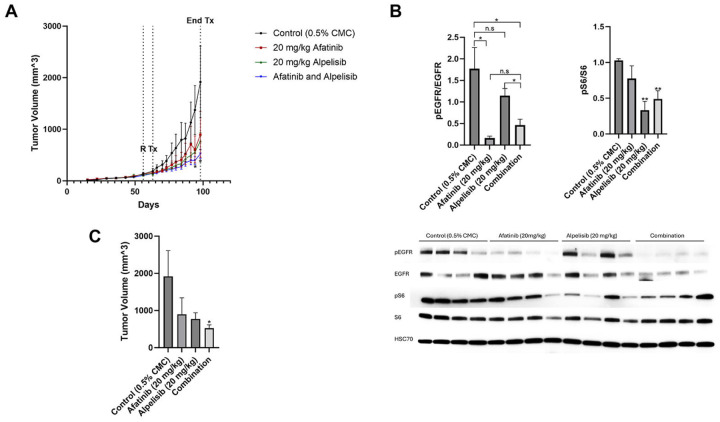
Dual EGFR/PI3K inhibition is most effective at reducing tumor volume *in vivo*. A) BT20 cells were injected subcutaneously into the mammary fat pad of nude mice. 56 days post implantation, when the average size was 100 mm^3^ , mice were randomized (R) to 15 mice into four groups and treated starting on day 63 post implantation (Tx) with control (0.5% carboxymethylcellulose), 20 mg/kg afatinib, 20 mg/kg alpelisib, or the combination of both afatinib and alpelisib Monday through Friday for 36 days. Statistical analysis was performed by Student’s t-test, and * indicates p<0.05 when comparing afatinib and alpelisib to control on day 94 and day 98. B) After four days of treatment, four mice from each group were euthanized and the protein from the tumors was collected and immunoblotted for pEGFR (Y1068), EGFR, pS6 (S235/236), S6, and HSC70 (loading control). Data represents the average of four mice from each group ±SEM and statistical analysis was performed by Student’s t-test, where ns indicates not statistically significant, * indicates p<0.05 and ** indicates p<0.01. C) Bar graph of data from A on day 98 (36 days of drug treatment). Statistical analysis was performed by Student’s ttest, where * indicates p<0.05 when compared to control. No other comparisons were statistically significant.

In a mammary fat pad xenograft model of BT20 cells in nude mice, mice were randomized 56 days post-implantation (indicated by R) when tumors reached 100 mm^3^, and treatment (indicated by Tx) was started 63 days post-implantation (Figure 7A). Mice were treated by oral gavage with control (0.5% carboxymethylcellulose), 20 mg/kg afatinib, 20 mg/kg alpelisib, or a combination of afatinib and alpelisib for 5 days a week (Monday through Friday). After the first four days of treatment, four mice from each group were sacrificed, and the tumors were harvested for protein analysis. Afatinib induced a statistically significant reduction in pEGFR when compared to controls and alpelisib induced a statistically significant reduction in pS6 compared to controls (Figure 7B). Afatanib induced a non-significant reduction in pS6 and alpelisib induced a non-significant reduction in pEGFR. Of note, the combination of afatinib and alpelisib did not further reduce downstream signaling as measured by pS6 compared to alpelisib alone.

Single agents alone reduced tumor volume after 36 days of treatment, however neither were statistically significant when compared to control due the slow and large variability in growth of the BT20 cells *in vivo* (Figure 7A and C). However, the reduction in tumor size by the combination of both afatinib and alpelisib were statistically significant when compared to the control. Drug treatment was terminated after 36 days of treatment due to the requirement of sacrificing control mice which had developed tumors larger than 20mm in any direction.

## Discussion

The goal of this study was to determine the incidence of EGFR amplification in breast cancer patients and to examine the benefit of molecularly targeted agents in models of EGFR amplification. Two cell lines with EGFR amplification and PI3K pathway mutations were compared to a control cell line with wild-type EGFR and PI3K signaling. EGFR and PI3K inhibitor combinations were tested on EGFR/PI3K signaling, cancer cell viability, cancer cell death, cell cycle, and mouse xenograft tumor growth.

We found EGFR amplification in approximately 1–2.5% of breast cancer patients with worse outcomes compared to patients without EGFR amplification (Figure 1). This EGFR amplification was enriched in TNBC, HER2 amplified, and ER negative patients. Importantly, TNBC patients are difficult to treat due to lack of defined molecular targets. Determination of targeted therapies aimed at a subset of breast cancer patients may provide therapeutic benefit in those who previously may have had poor prognosis and could help shift the paradigm from treatment of large populations with general chemotherapy, to treating smaller populations with a focused and specific molecular inhibition strategy to provide clinical benefit. This study provides evidence that dual EGFR and PI3K inhibition in TNBC with EGFR amplification and PI3K aberrations act synergistically in reducing cell viability, as well as inducing apoptosis *in vitro*, and reducing tumor growth *in vivo*. Further, we provide evidence of a patient population which can be determined by molecular profiling and would benefit from treatment with this therapy. For example, of the 272 EGFR amplified patients in the Caris dataset, only three of them received the EGFR inhibitor Neratinib. This study presents evidence for potential efficacy of EGFR and PI3K inhibitors in these patients, and patients are currently not receiving this type of therapy in the clinic. This warrants clinical trial investigation into whether these patients would benefit from this therapy.

It is interesting that a higher percentage of patients with EGFR amplification in TNBC (2.45%) do not have an altered survival (Figure 1E and Additional File 1 A). This likely reflects that TNBC or Basal tumors generally have a poor prognosis. None-the-less, our data using cell models of EGFR amplification and PI3K pathway mutations suggest that these patients may benefit from targeted EGFR and PI3K pathway inhibition. Further, this study characterized a model looking at TNBC, however considering HER2 enriched had a trend of worse OS, and ER+HER2− patients had a statistically significantly worse OS, there are likely other breast cancer patient populations with EGFR amplification and PI3K alteration who can benefit from this dual therapy (Additional File 1).

The two cell lines with EGFR amplification and PI3K alterations (BT20 and MDA-MB-468) exhibited significant inhibition of downstream signaling with the combination of EGFR inhibitors and PI3K inhibitors, however the MDA-MB-231 cells without the alterations were less affected by the drug combination (Figure 4, Additional File 3). Further, the combination of drugs had a synergistic effect on cell viability and cell cycle arrest of the cell lines with EGFR amplification and PI3K alterations (Figure 5 and 6, Additional File 5). In cells with EGFR amplification and PI3K mutation, the combination of EGFR and PI3K inhibitors induced apoptosis (Figure 6D and E, Additional File 6). Lastly, in a xenograft mouse model, the combination of EGFR and PI3K inhibitors reduced downstream signaling in the EGFR and PI3K pathways and inhibited the tumor growth in a statistically significant manner when compared to vehicle control. Either drug alone did not reduce the tumor volume in a statistically significant manner, indicating that the most effective treatment for these tumors with EGFR amplification and PI3K mutation is the combination of both an EGFR inhibitor and PI3K inhibitor (Figure 7).

Between 20–50% of breast cancers exhibit PIK3CA mutations, with the most common being hormone receptor positive or HER2 amplified breast cancer (48–53). It is noteworthy that PIK3CA alterations found in this study (36–39%) in EGFR amplified patients is similar to the percentage observed in HER2 amplified patients (30%) (51–53). Combining HER2 targeted therapy with PI3K inhibitors has been previously studied, and our study presents evidence that there is a similar population of EGFR amplified patients who could benefit from combination therapy (54, 55). Depending on the dataset, this study identified PI3K pathway alterations in 54–71% of patients with EGFR amplification (Figure 3).

Alpelisib, which targets PIK3CA, has been FDA approved for treatment in hormone receptor positive breast cancer with PIK3CA mutations in combination with endocrine therapy, as it significantly improved progression-free survival (56–58). In TNBC, alpelisib monotherapy showed no overall response rate or clinical benefit rate in patients with PI3K alterations, and the trial recruitment was ended due to lack of efficacy (53). Therefore, alpelisib may benefit from combination with other targeted therapies in breast cancer patients, especially in TNBC. Capivasertib, which targets AKT, has been approved for treatment of metastatic breast cancers in combination with ER targeted therapies, and improves patient survival (59, 60),

EGFR and PI3K inhibitor combination has been studied in NSLC, head and neck squamous cell carcinoma (HNSCC), glioblastoma, ovarian cancer and breast cancer, however these studies did not monitor EGFR amplification or PI3K pathway mutation, and did not use EGFR amplified and PI3K mutated cell lines as models (61–69). Further, a recent study looking at PDX models with PI3K mutations found through a high throughput synergy model that afatinib and alpelisib were synergistic, which confirms our data, however they also did not monitor EGFR status (70). Our study indicates that it is important to determine the subset of patients who would benefit from this combination, by stratifying patients with EGFR amplification and PI3K pathway alterations. We also do not rule out the possibility of combining these drugs with HER2 targeted therapies, as we found that up to 5.8% of EGFR amplification is found in breast cancer patients with HER2 amplification or HER2 enrichment (Figure 1). In fact, there is a study showing synergy with BKM120 and the HER2 inhibitor Trastuzumab, which further provides evidence that combining PI3K inhibitors with EGFR family inhibitors may provide clinical benefit in patients with HER2 and EGFR amplification and PI3K mutation (71).

We studied multiple EGFR (erlotinib or afatinib) and PI3K (BKM120 or alpelisib) inhibitors (Figures 4, 5, 6, 7 and Additional Files 3, 5 and 6). It is important to note that afatinib inhibits wild-type (WT) EGFR at the lowest IC50, and then at increasing IC50s it inhibits L858R EGFR, HER2 and HER4 (72, 73), and BKM120 also inhibits mTOR, VPS34 and DNAPK (74). Alpelisib is a more specific PI3K inhibitor, without observed off target effects. Since we tested different EGFR and PI3K inhibitors and observed similar reductions in downstream signaling, cell viability, cell cycle, and apoptosis when the drugs were combined, it is likely that the observed effects were due to a class effect of the drugs. Taken together with the *in vivo* data in this study, drugs from these classes warrant investigation in combination in breast cancer patients who exhibit EGFR amplification and PI3K alterations.

Dual EGFR and PI3K inhibition *in vitro* exhibited significant synergy in this study, however the *in vivo* data was more modest. The modest effects are likely due to the short half-life of alpelisib (approximately 1.5 hours in mice), whereas afatinib has a longer half-life (approximately 21 hours in mice) (75, 76). Compared to the *in vitro* work where cells were treated continuously for 48–72 hours with the drugs, this could account for the difference in observed results when comparing *in vitro* and *in vivo* results in this study. The half-life of afatinib in human patients is approximately 30–40 hours and alpelisib is approximately 7.5 hours, suggesting with the daily dosing used, there would be better steady state levels than in the mouse (77, 78).

## Conclusions

Approximately 1–2.5% of breast cancer patients have EGFR amplification, with a worse prognosis. Up to 71% of the EGFR amplified population exhibits co-incidence of PI3K pathway activating alterations. Dual targeting of these pathways in cells which express EGFR amplification and PI3K alteration with chemical inhibitors demonstrated synergy *in vitro*, and their combination reduced tumor growth in a xenograft model more than either drug alone when compared to control. Although rare, our data suggests that breast cancers that exhibit EGFR amplification and PI3K pathway alterations are a poor prognosis and should be approached with targeted therapy similar to those with HER2 amplification. Taken together, this study demonstrated that the combination of EGFR and PI3K pathway inhibitors should be tested in breast cancer patients with EGFR amplification and PI3K pathway alteration. This will shift the paradigm for the treatment regimen in this patient population, especially triple negative breast cancer patients with fewer targeted therapy options.

## Supplementary Material

Supplement 1 Additional File 6
File Format: .tiff
Title of Data: Dual EGFR/PI3K inhibition induce apoptosis in EGFR amplified and PI3K altered TNBC.
Description of Data: A) MDA-MB-231, B) MDA-MB-468 and C) BT20 cells were pretreated with 20 μM Z-VAD-FMK (ZVAD) for one hour, and then were treated with DMSO, 10 μM erlotinib, 1 μM BKM120 or the combination of both, with continued ZVAD treatment for 48 hours. Dead cell percentage was calculated by using CytoTox-Glo by Promega. Data represents the average ±SEM of at least three independent experiments. Statistical analysis was performed by One-way ANOVA, with ns indicating not statistically significant, * indicating p<0.05, ** indicating p<0.01 and *** indicating p<0.001.

Supplement 2 Additional File 1
File Format: .tiff
Title of Data: Overall survival of patients with EGFR amplification by subtype.
Description of Data: Using the Caris dataset, overall survival of breast cancer patients with EGFR amplification (red) compared to no amplification (blue) in A) Triple negative breast cancer, B) Basal, C) HER2 enriched, or D) ER+ HER2−.

Supplement 3 Additional File 2
File Format: .tiff
Title of Data: Oncoplot for PI3K pathway alterations in EGFR amplified breast cancer.
Description of Data: Patient data from cbioportal.org plotting varying mutations or amplifications by gene in the PI3K pathway.

Supplement 4 Additional File 3
File Format: .tiff
Title of Data: EGFR/PI3K dual inhibition significantly reduce downstream signaling in EGFR amplified and PI3K altered TNBC.
Description of Data: A) MDA-MB-468 and BT20 cells were starved in serum-free RPM1 media for 1 hour with 0, 1 or 10 μM erlotinib, and then stimulated with or without 25 ng/mL EGF for 10 minutes. Cells were lysed and immunoblotted for pEGFR (Y1068) and EGFR (loading control). B) Darker exposure for MDA-MB-231 panel from Figure 4B which was used for quantification. C) MDA-MB-231, MDA-MB-468 and BT20 cells were treated with 10 μM erlotinib, 1 μM BKM120 or a combination of both for 24 hours, and were then lysed and immunoblotted for pAKT (S473), AKT, pMAPK (T202/Y204), ERK2, p-p70S6K (T389), p70S6K and HSC70 (loading control). In at least three independent experiments, the band density of the phosphorylated protein relative to total protein was averaged ±SEM for D) pMAPK/ERK2, E) pAKT/AKT and F) p-p70S6K/p70S6K. Student’s t-test was performed, where ns indicates not statistically significant, * indicates p<0.05, ** indicates p<0.01, *** indicates p<0.001 and **** indicates p<0.0001.

Supplement 5 Additional File 4
File Format: .tiff
Title of Data: Erlotinib treatment does not activate pHER3.
Description of Data: MDA-MB-231, MDA-MB-468 and BT20 cells were treated with 10 μM erlotinib, 1 μM BKM120 or the combination of both for 24 hours before the cells were lysed and immunoblotted for pHER3 (Y1289) and HER3 (loading control).

Supplement 6 Additional File 5
File Format: .tiff
Title of Data: EGFR/PI3K inhibition reduces viability, induces cell cycle arrest in TNBC with EGFR amplification, PI3K alteration.
Description of Data: A) MDA-MB-231, B) MDA-MB-468 and C) BT20 cells were treated with the indicated doses for 48 hours, and then viability was determined by Promega CellTiter-Glo 2.0. Data represents the average ±SEM of at least three independent experiments, and statistical analysis was performed as Two-Way ANOVA, and the p value indicates interaction, where ns indicates not statistically significant, * indicates p<0.05, *** indicates p<0.001 and **** indicates p<0.0001. D) MDA-MB-231, E) MDA-MB-468 and F) BT20 cells were treated with DMSO control, 1 μM erlotinib or 10 μM BKM120 or the combination of erlotinib and BKM120 for 48 hours, and then were harvested, washed, fixed and stained with propidium iodide before being analyzed by flow cytometry and FlowJo for cell cycle changes. Data represents the average ±SEM of at least three independent experiments, and Two-Way ANOVA statistical analysis was performed where * indicates p<0.05, ** indicates p<0.01 and *** indicates p<0.001.

## Data Availability

The MSKCC, TCGA and Metabric datasets analyzed in this study are available on cBioPortal.org (35–37). The Caris deidentified sequencing data are owned by Caris Life Sciences and cannot be publicly shared due to the data usage agreement in place. These data will be made available to researchers for replication and verification purposes through our letter of intent process, which are generally fulfilled within 6 months. For more information on how to access this data, please contact Joanne Xiu at jxiu@carisls.com. All other data generated in this study are included in this published article and its supplementary information.

## List of Abbreviations

EGFR: Epidermal Growth Factor Receptor
PI3K: Phosphoinositide 3-kinase
TNBC: Triple Negative Breast Cancer
FISH: fluorescent in situ hybridization
PI: propidium iodide
ER: Estrogen Receptor
HER2: Human epidermal growth factor receptor 2
mTOR: mechanistic target of rapamycin
AKT: Protein Kinase B
EGF: epidermal growth factor
MAPK: mitogen activated protein kinases
TKI: tyrosine kinase inhibitor
P70S6K: ribosomal protein S6 kinase beta 1
SEM: standard error of the mean
TCGA: The Cancer Genome Atlas Program
MSKCC: Memorial Sloan Kettering Cancer Center
IDC: invasive ductal carcinoma
ILC: Invasive lobular carcinoma
OS: overall survival
PTEN: phosphatase and tensin homolog
DMSO: dimethyl sulfoxide
NSCLC: non-small cell lung cancer
HNSCC: head and neck squamous cell carcinoma
PDX: patient derived xenograft
WT: wild-type
DNAPK: DNA-dependent protein kinase
