# Supplementary figures and images for: EGFR amplification and PI3K pathway mutations identify a subset of breast cancers that synergistically respond to EGFR and PI3K inhibition
Source: bioRxiv. 2025 Sep 16:2025.06.03.657674. Originally published 2025 Jun 7. Preprint. [Version 2] doi: 10.1101/2025.06.03.657674 (PMC12157506; doi:10.1101/2025.06.03.657674)

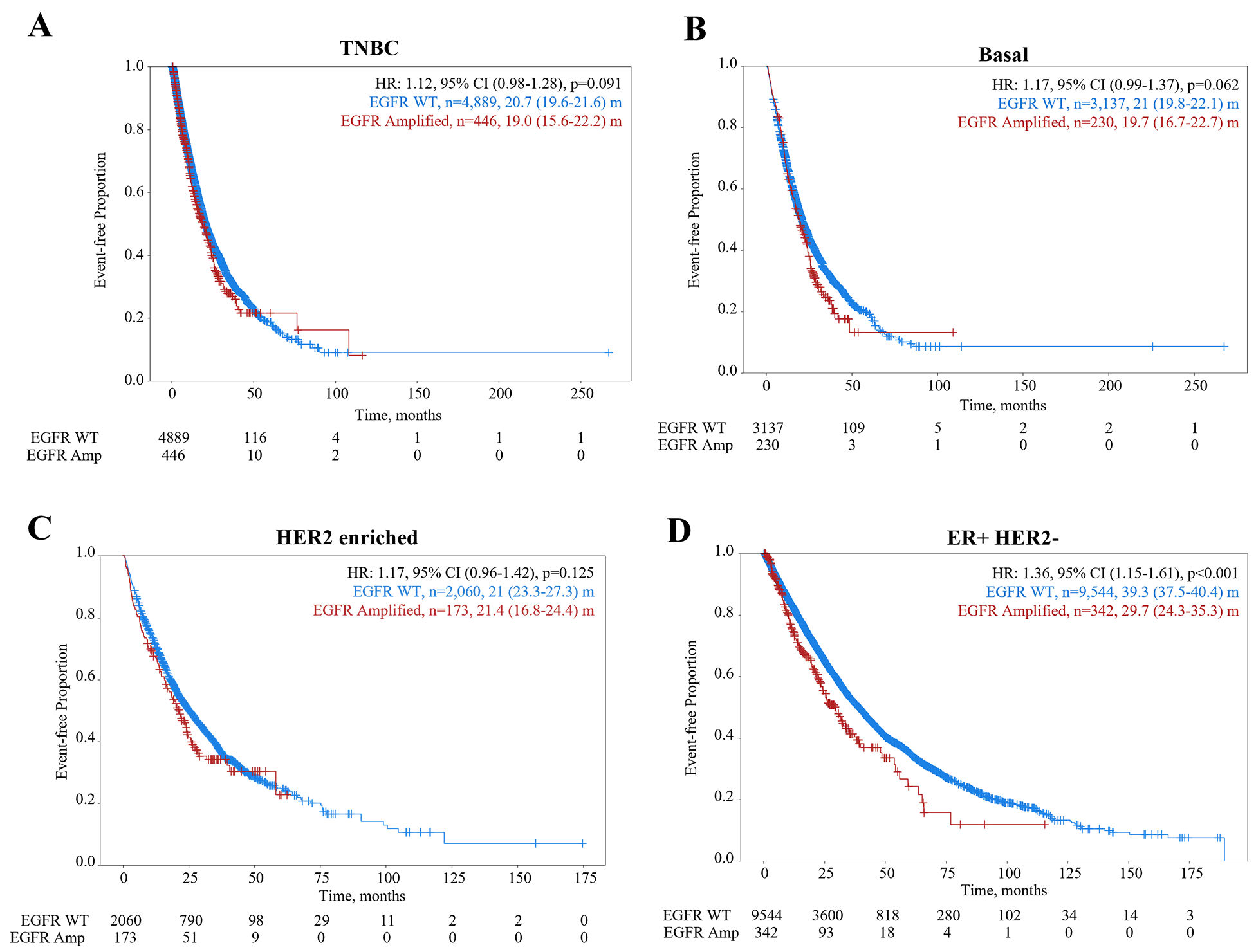

Supplement: Supplement 1 — Figure S1 Overall survival of patients with EGFR amplification by subtype. Using the Caris dataset, overall survival of breast cancer patients with EGFR amplification (red) vs WT (blue) in A) Triple negative breast cancer, B) Basal, C) HER2 enriched, or D) ER+ HER2−. [file media-1.tif]

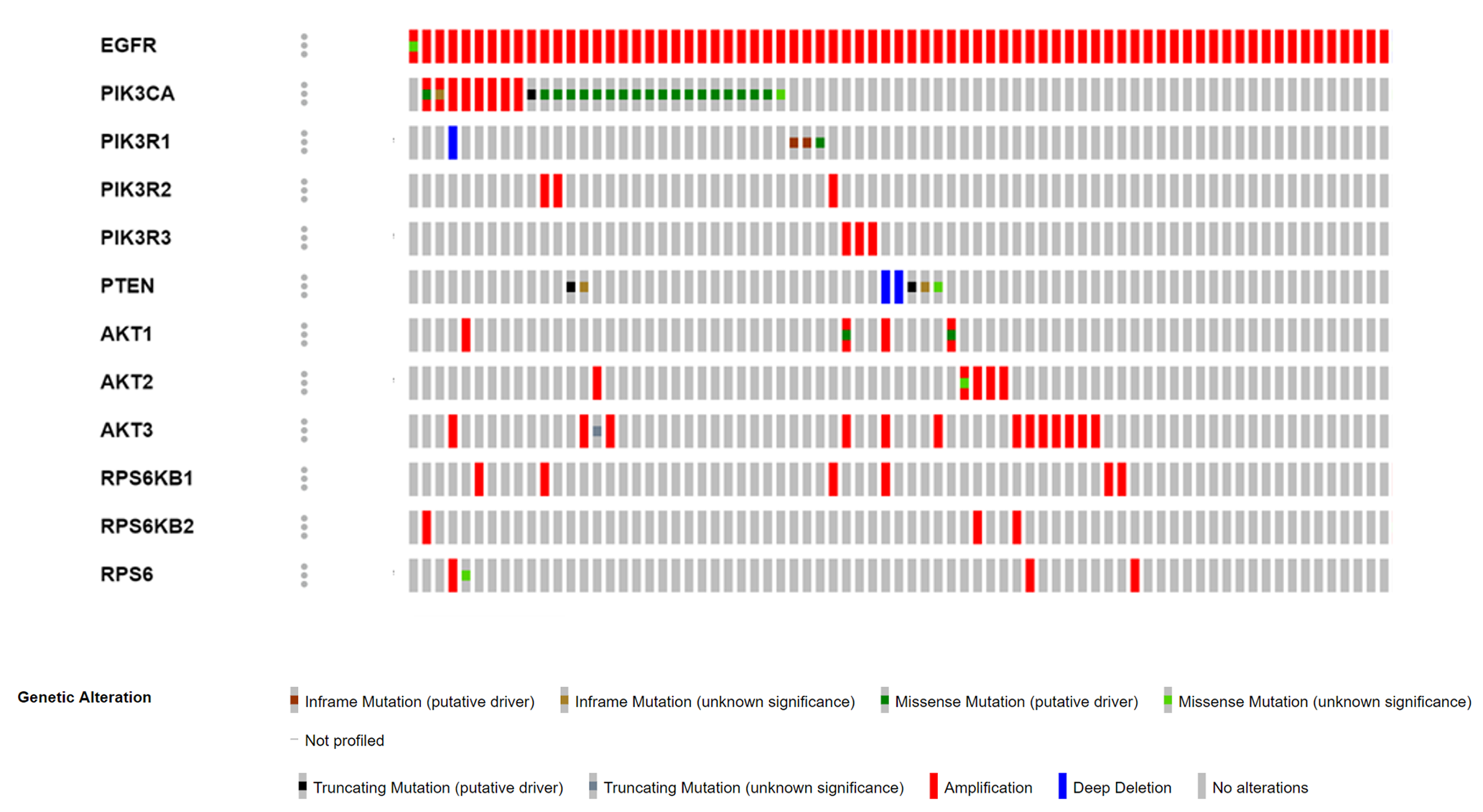

Supplement: Supplement 2 — Figure S2 Oncoplot for PI3K pathway alterations in EGFR amplified breast cancer. Patient data from cbioportal.org plotting varying mutations or amplifications by gene in the PI3K pathway. [file media-2.tif]

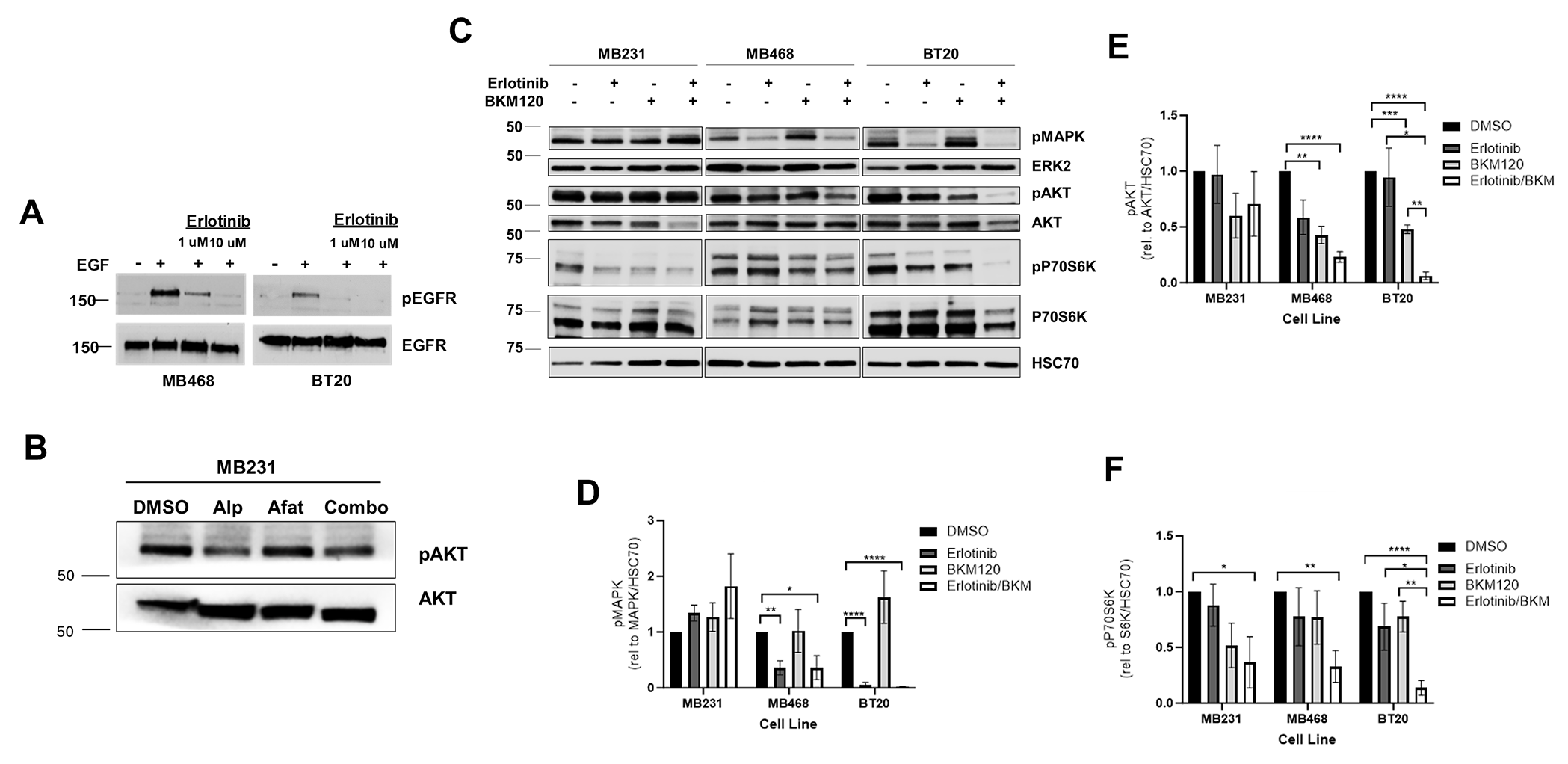

Supplement: Supplement 3 — Figure S3 EGFR/PI3K dual inhibition significantly reduce downstream signaling in EGFR amplified and PI3K altered TNBC. A) MDA-MB-468 and BT20 cells were starved in serum-free RPM1 media for 1 hour with 0, 1 or 10 μM erlotinib, and then stimulated with or without 25 ng/mL EGF for 10 minutes. Cells were lysed and immunoblotted for pEGFR (Y1068) and EGFR (loading control). B) Darker exposure for MDA-MB-231 panel from Figure 4B which was used for quantification. C) MDA-MB-231, MDA-MB-468 and BT20 cells were treated with 10 μM erlotinib, 1 μM BKM120 or a combination of both for 24 hours, and were then lysed and immunoblotted for pAKT (S473), AKT, pMAPK (T202/Y204), ERK2, p-P70S6K (T389), P70S6K and HSC70 (loading control). In three independent experiments, the band density of the phosphorylated protein relative to total protein was averaged ±SEM for D) pMAPK/ERK2, E) pAKT/AKT and F) p-P70S6K/P70S6K. Student’s t-test was performed, where ns indicates not statistically significant, * indicates p<0.05, ** indicates p<0.01, *** indicates p<0.001 and **** indicates p<0.0001. [file media-3.tif]

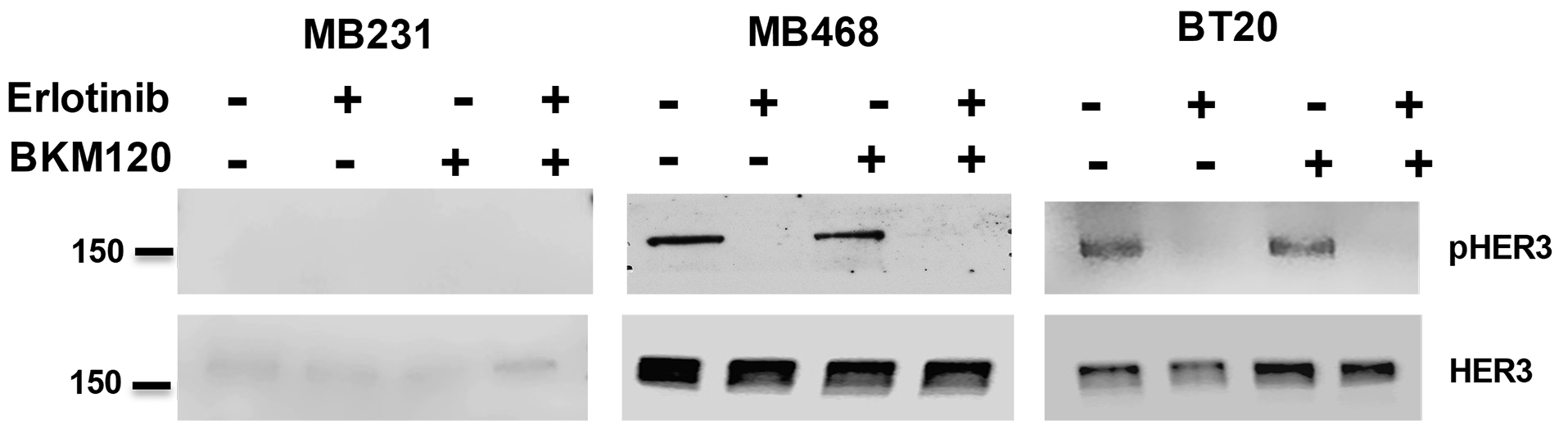

Supplement: Supplement 4 — Figure S4 Erlotinib treatment does not activate pHER3. MDA-MB-231, MDA-MB-468 and BT20 cells were treated with 10 μM erlotinib, 1 μM BKM120 or the combination of both for 24 hours before the cells were lysed and immunoblotted for pHER3 (Y1289) and HER3 (loading control). [file media-4.tif]

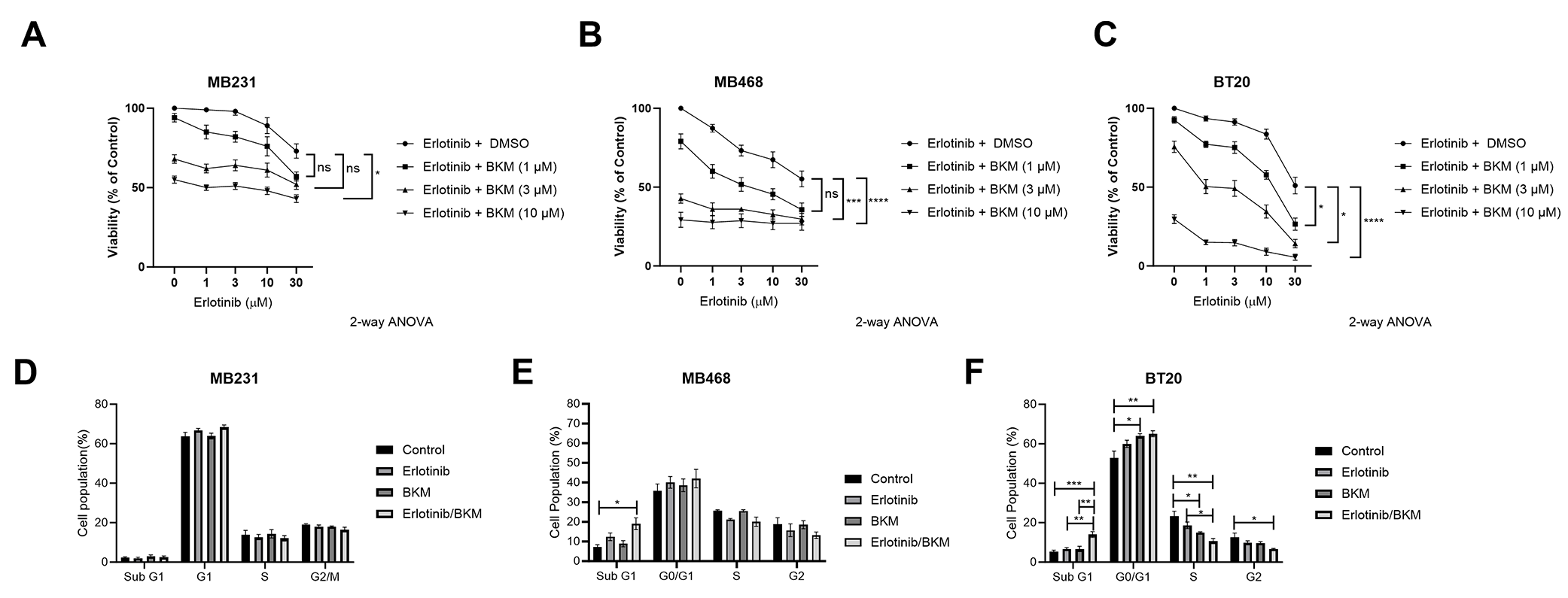

Supplement: Supplement 5 — Figure S5 EGFR/PI3K inhibition reduces viability, induces cell cycle arrest in TNBC with EGFR amplification, PI3K alteration. A) MDA-MB-231, B) MDA-MB-468 and C) BT20 cells were treated with the indicated doses for 48 hours, and then viability was determined by Promega CellTiter-Glo 2.0. Data represents the average ±SEM of three independent experiments, and statistical analysis was performed as Two-Way ANOVA, and the p value indicates interaction, where ns indicates not statistically significant, * indicates p<0.05, *** indicates p<0.001 and **** indicates p<0.0001. D) MDA-MB-231, E) MDA-MB-468 and F) BT20 cells were treated with DMSO control, 1 μM erlotinib or 10 μM BKM120 or the combination of erlotinib and BKM120 for 48 hours, and then were harvested, washed, fixed and stained with propidium iodide before being analyzed by flow cytometry and FlowJo for cell cycle changes. Data represents the average ±SEM of at least three independent experiments, and Two-Way ANOVA statistical analysis was performed where * indicates p<0.05, ** indicates p<0.01 and *** indicates p<0.001. [file media-5.tif]

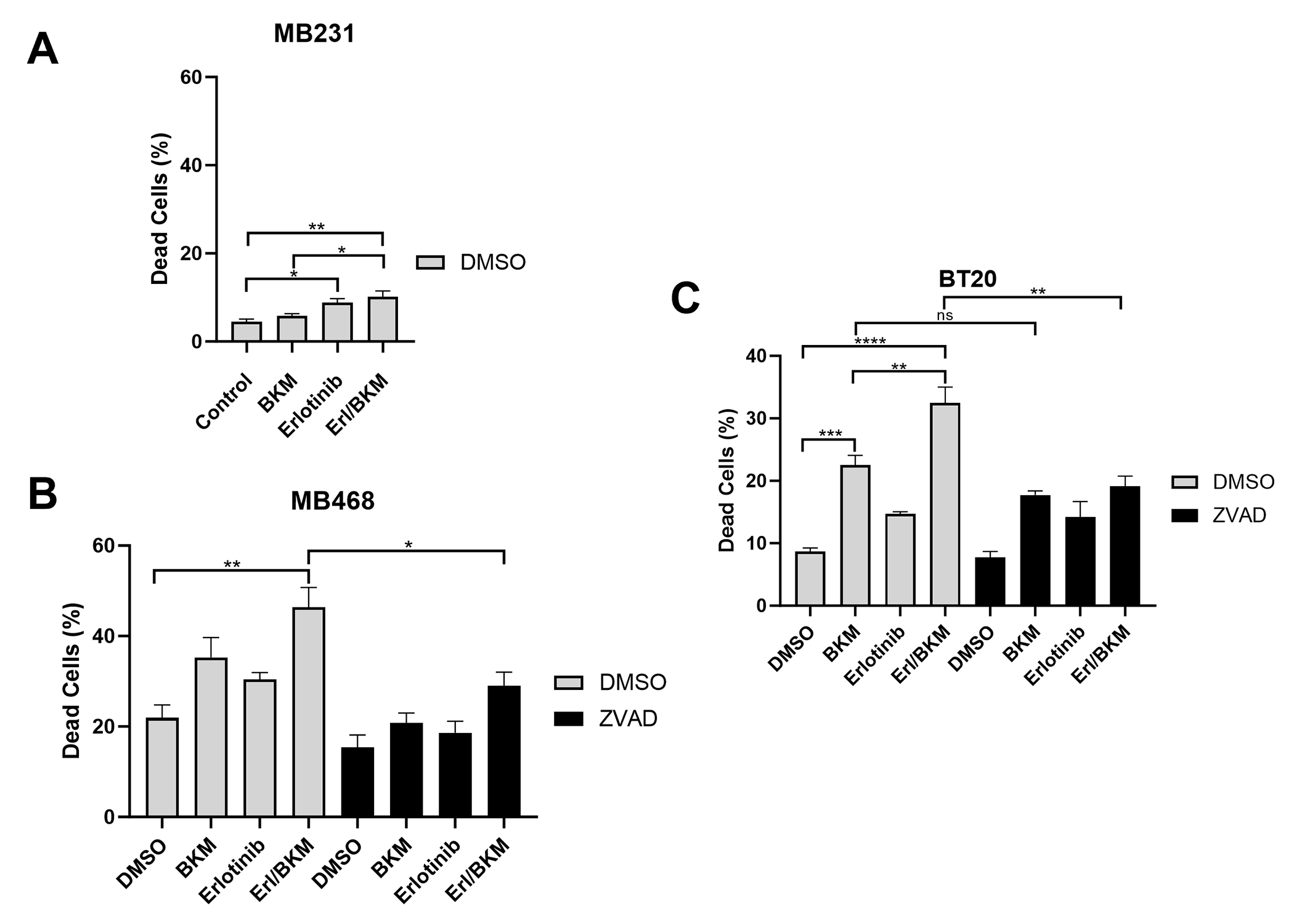

Supplement: Supplement 6 — Figure S6 Dual EGFR/PI3K inhibition induce apoptosis in EGFR amplified and PI3K altered TNBC. A) MDA-MB-231, B) MDA-MB-468 and C) BT20 cells were pretreated with 20 μM Z-VAD-FMK (ZVAD) for one hour, and then were treated with DMSO, 10 μM erlotinib, 1 μM BKM120 or the combination of both, with continued ZVAD treatment for 48 hours. Dead cell percentage was calculated by using CytoTox-Glo by Promega. Data represents the average ±SEM of three independent experiments. Statistical analysis was performed by One-way ANOVA, with ns indicating not statistically significant, * indicating p<0.05, ** indicating p<0.01 and *** indicating p<0.001. [file media-6.tif]
